# Charge carrier transport in perylene-based and pyrene-based columnar liquid crystals

**DOI:** 10.3762/bjoc.19.128

**Published:** 2023-11-16

**Authors:** Alessandro L Alves, Simone V Bernardino, Carlos H Stadtlober, Edivandro Girotto, Giliandro Farias, Rodney M do Nascimento, Sergio F Curcio, Thiago Cazati, Marta E R Dotto, Juliana Eccher, Leonardo N Furini, Hugo Gallardo, Harald Bock, Ivan H Bechtold

**Affiliations:** 1 Departamento de Física, Universidade Federal de Santa Catarina, Florianópolis 88040-900, SC, Brazil; 2 Departamento de Química, Universidade Federal de Santa Catarina, Florianópolis 88040-900, SC, Brazil; 3 Departamento de Fisica, Universidade Federal de Ouro Preto, Ouro Preto 35400-000, MG, Brazil; 4 Centre de Recherche Paul Pascal, CNRS, 115 av. Schweitzer, 33600 Pessac, France

**Keywords:** charge carrier transport, columnar liquid crystal, organic electronics, perylene, pyrene

## Abstract

Electron and hole transport characteristics were evaluated for perylene-based and pyrene-based compounds using electron-only and hole-only devices. The perylene presented a columnar hexagonal liquid crystal phase at room temperature with strong molecular π-stacking inside the columns. The pyrene crystallizes bellow 166 °C, preserving the close-packed columnar rectangular structure of the mesophase. Photophysical analysis and numerical calculations assisted the interpretation of positive and negative charge carrier mobilities obtained from fitting the space charge limited regime of current vs voltage curves. The pyrene-based material demonstrated an electron mobility two orders of magnitude higher than the perylene one, indicating the potential of this class of materials as electron transporting layer.

## Introduction

Conjugated organic molecules have been widely investigated due to their interesting transport properties and promising applications as active layer in organic photovoltaics (OPVs), organic field effect transistors (OFETs), organic light-emitting diodes (OLEDs) and sensors [1–2]. Columnar liquid crystals are attractive due to their solution processability and their self-organization in highly anisotropic supramolecular architectures, which favors the mainly one-dimensional migration of charge carriers with an anisotropy of the charge carrier mobility (parallel vs perpendicular to the columnar axis) of up to ten orders of magnitude [3–7]. In 1994, Adam and collaborators obtained a hole-mobility of up to 0.1 cm^2^ V^−1^ s^−1^ for a triphenylene-based columnar liquid crystal [8], motivating intense research activity to understanding charge transport in columnar mesophases [9–14]. High charge carrier mobilities of 1.1 cm^2^ V^−1^ s^−1^ for p-type and up to 6.0 cm^2^ V^−1^ s^−1^ for n-type liquid-crystalline semiconductors have already been reported [15].

Among the various discotic liquid crystal materials, perylene derivatives are among the most investigated due to their easy functionalization, high chemical and thermal stability, strong photoluminescence, and n-type semiconductor character. They tend to adopt columnar organization due to the strong π–π interaction of the rigid cores, providing a path for the efficient conduction of electrons [16]. Perylene diimide derivatives display good electronic mobilities (10^−3^ to 10^−1^ cm^2^ V^−1^ s^−1^) and are considered suitable electron acceptors for photovoltaic applications [17–20]. Pyrene derivatives have also been widely investigated in recent decades. They exhibit excellent photoelectric properties such as strong emission, efficient excimer formation, and suitable intermolecular stacking for good charge carrier transport. They have been applied as active layer in OFETs with high ambipolar mobility due to their well-defined monocrystalline microstructures resulting from strong π–π interactions [7,21–24].

In this work, we investigated a perylene and a pyrene-based columnar liquid crystal in hole-only and electron-only devices to compare their charge carriying properties. The molecular organization and photophysical performances are coherent with the charge transport behavior. DFT calculations assisted the interpretation of electron and hole migration mechanism using the frontier orbital energies and the conjugation within the π-system.

## Results and Discussion

**Scheme 1:**
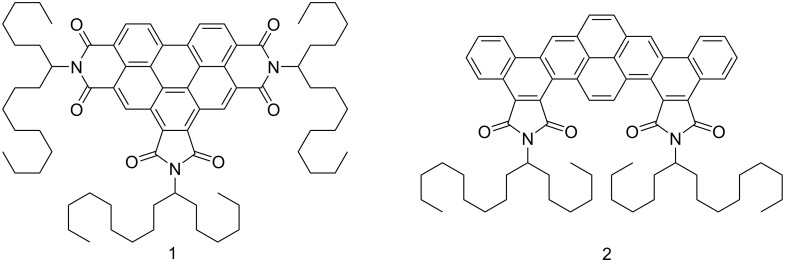
Molecular structures of compounds **1** and **2**.

The syntheses of **1** and **2** were previously published in [25] and [26], respectively. **1** is a benzo[*ghi*]perylene-hexacarboxylic trialkylimide and **2** a dinaphtho[2,1-*a*;1,2-*i*]pyrene-tetracarboxylic dialkylimide, both with asymmetrically branched alkyl swallow-tails derived from 7-aminohexadecane (Scheme 1 illustrates their molecular structure). In Table 1, we show the thermal characteristics and observed mesophases. Compound **1** presents a wide range columnar hexagonal phase (Col_hex_) preserved at room temperature by cooling from the isotropic. Compound **2** shows an additional columnar rectangular phase (Col_rect_) below the Col_hex_ and crystallizes under 166 °C. The HOMO and LUMO energy levels are also given in Table 1.

**Table 1:** Characteristics of **1** and **2** extracted from [17] and [18], respectively.

Comp.	Phase sequence (°C)^a^	HOMO (eV)	LUMO (eV)

**1**	Col_hex_ – 177 - Iso	−6.62	−3.79
**2**	Cr – 166 – Col_rect_ – 225 – Col_hex_ – 248 – Iso	−5.65	−3.32

^a^Determined by DSC, XRD and POM.

**Figure 1:**
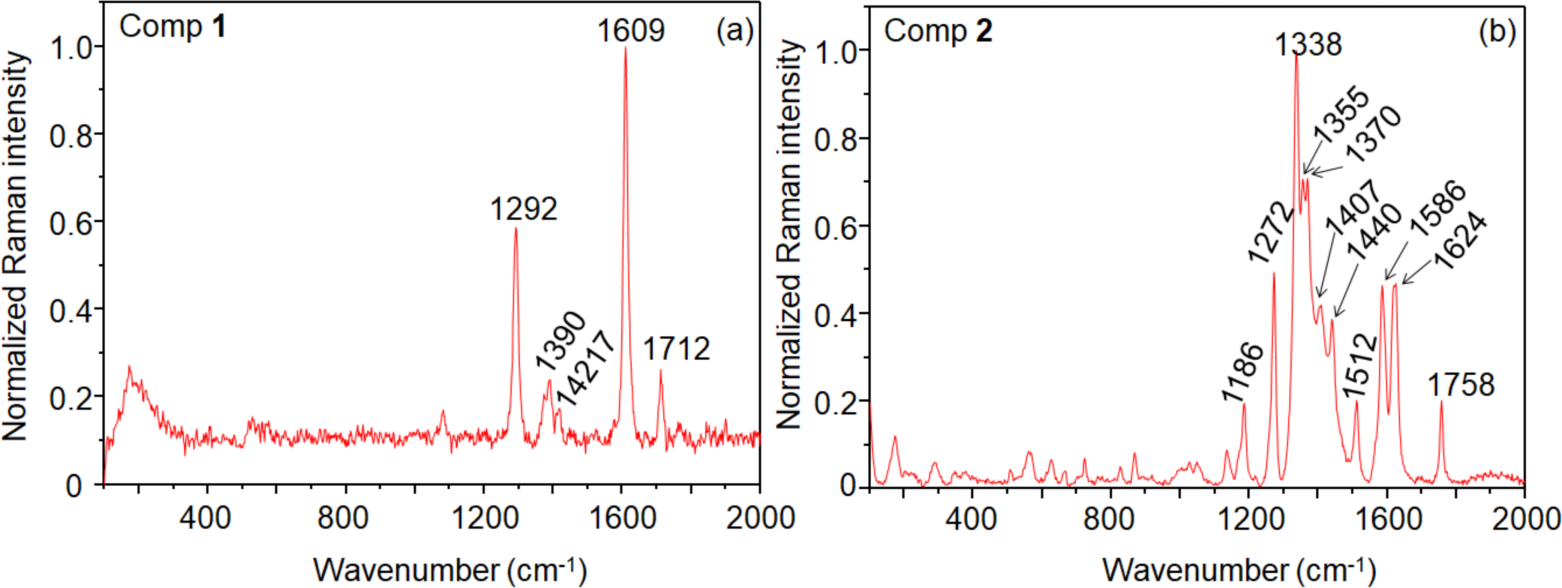
Raman spectra of **1** (a) and **2** (b) in powder.

Raman spectra of both compounds were acquired off-resonance (Figure 1). Compound **1** presents the main peak at 1609 cm^−1^ assigned to C=C stretching from the chromophore, a peak of intermediate intensity at 1292 cm^−1^ assigned to C–H bending and ring stretching, and a less intense peak at 1712 cm^−1^ which corresponds to C=O stretching [27]. The pair at 1390 and 1417 cm^−1^ is attributed to perylene ring stretching [28]. As observed for bis(phenethylimido)perylene (PhPTCD) [29] only a small number of peaks is observed. In contrast, compound **2** presents a larger number of peaks, with the spectrum being dominated by the peak at 1338 cm^−1^ (C–N stretching). The peak at 1272 (C–H bending and ring stretching), and the pair of peaks at 1586 and 1624 cm^−1^ are assigned to the C=C stretching mode [30]. Less intense peaks can be observed at 1186 (C–H bending), 1512 (perylene ring stretching), and 1758 cm^−1^ (C=O).

**Figure 2:**
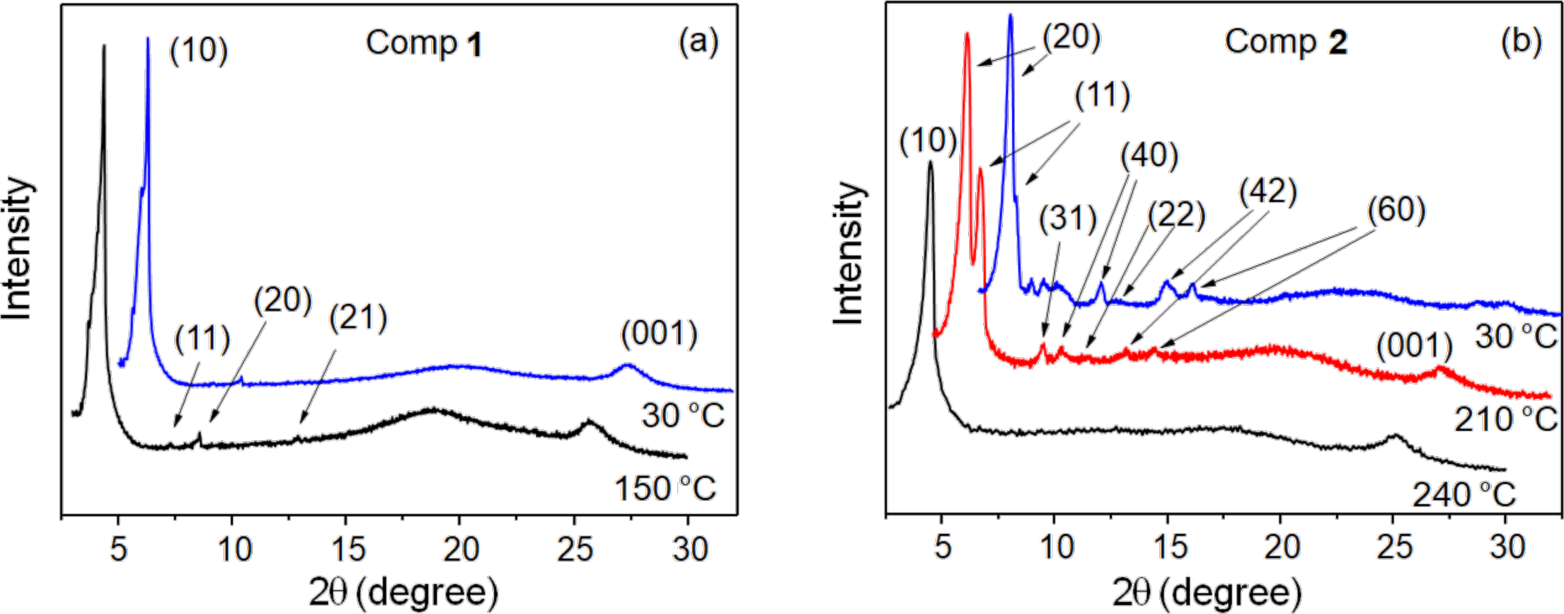
XRD measurements of **1** (a) and **2** (b) captured on cooling from the isotropic phase, indicating the Miller indices.

X-ray diffraction (XRD) measurements of **1** and **2** are shown in Figure 2. The Miller indices indicate the Col_hex_ and Col_rect_ character of the mesophases [31]. Despite crystallization of **2**, the Col_rect_ order is partially preserved at room temperature. The Col_hex_ lattice parameter (a) obtained is 23.7 Å for **1** and 22.9 Å for **2**. The *a*/*b* aspect ratio of the Col_rect_ phase of **2** is equal to 2.08, representing an elongation of around 20% concerning the hexagonal mesophase (where *a*/*b* = 1.73). The π-stacking distance between neighboring disks inside the columns, indicated by the (001) peak, is ≈3.5 Å for all mesophases.

**Figure 3:**
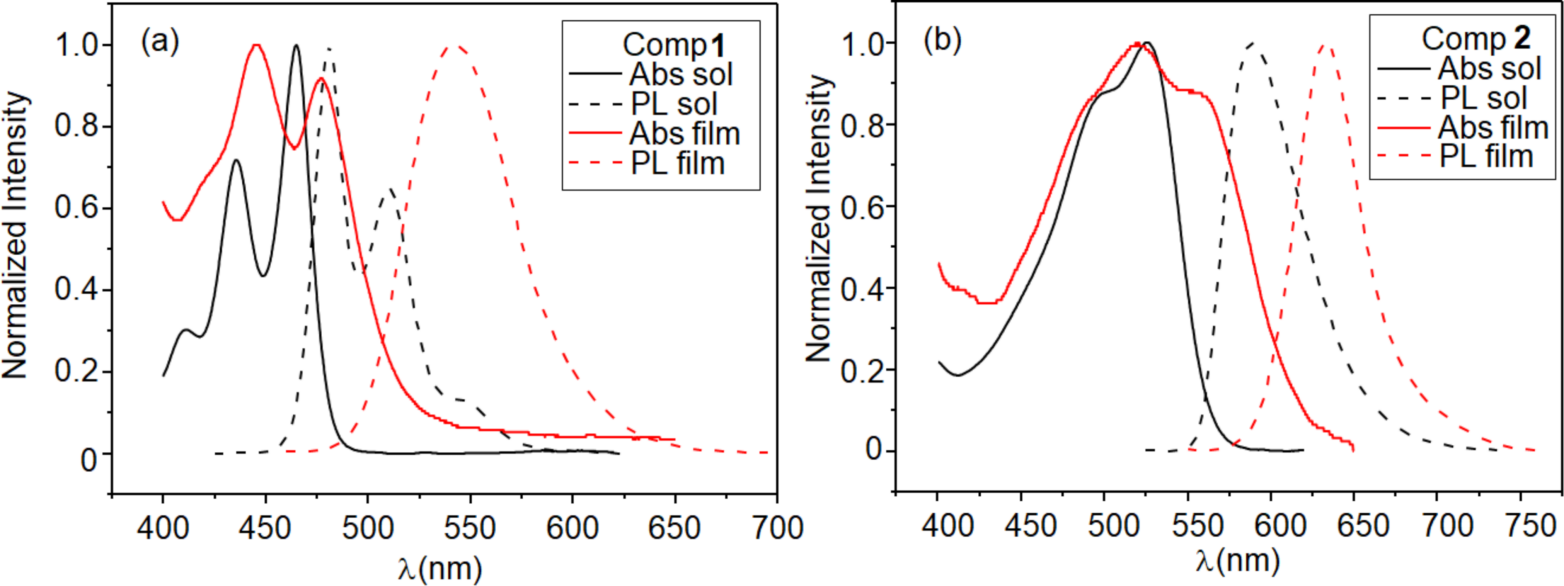
Absorption and PL in chloroform solution and in spin-coated films for compounds **1** (a) and **2** (b). The PL was excited at the wavelength of maximum absorption.

Absorption and photoluminescence (PL) of **1** and **2** are presented in Figure 3 for solution and spin-coated film. Compound **1** shows the three well-defined bands typical of perylene absorption and PL in solution. The absorption becomes broader and the PL destructured and red-shifted in the film. These results indicate molecular π-stacking aggregation and excimer formation on the films [2].

**Figure 4:**
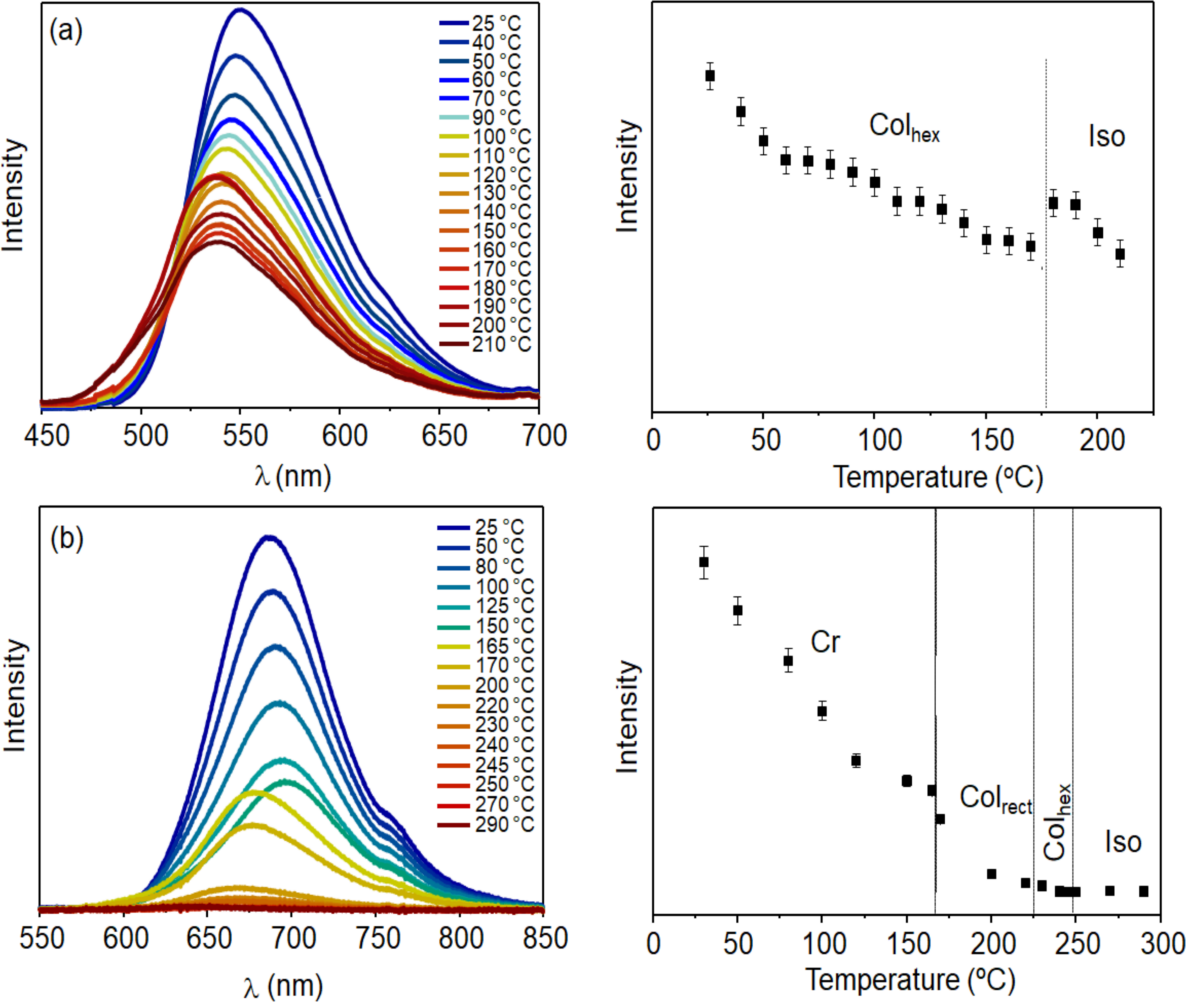
PL as a function of temperature for **1** (a) and **2** (b) casting films on heating. Left: PL spectra; right: intensity of the PL maximum. The PL was excited at the wavelength of maximum absorption.

The PL in film was obtained as a function of temperature (Figure 4). The observed reduction of the PL intensity on heating is expected for organic semiconductors due to self-quenching aggregates and nonradiative decay processes that are thermally activated [32]. However, an increase in the emission intensity can be observed for **1** at the Col_hex_–Iso transition. The stronger π-stacking aggregates present in the Col_hex_ of **1**, reduce the emission compared to the the disordered isotropic phase. The enhanced and blue-shifted emission at the transition to the isotropic phase arise from the excited isolated molecules, where the exciton decays radiatively without diffusion. For the isolated molecules, the more localized π-orbital results in a higher energy emission state. The same effect was previously observed for another perylene-based ColLC compound, indicating the strong molecular interaction between neighboring molecules inside the columnar structure [33]. Due to the high temperature of the Iso phase for **2**, the PL intensity almost vanished. However, a slight increase could also be observed at the Col_hex_–Iso transition. The reduction of PL at the Cr–Col_rect_ transition also reflects stronger molecular π-stacking of **2** in the Col_rect_ phase compared to the crystalline state.

The excited state lifetimes of **1** and **2** in chloroform solutions and spin-coated films, both excited at 401 nm, are listed in Table 2. The fitted fluorescence decay curves are shown in Supporting Information File 1 (Figure S1). For diluted solutions of **1** and **2**, monoexponential lifetimes in the nanosecond timescale were observed and attributed to emission from monomeric species (6.54 and 4.14 ns, respectively). In the spin-coated films, the best fitting of the decay curves of **1** and **2** indicated three and two lifetimes, respectively. Compound **1** displayed a dominant and longer lifetime compared to solution, 25.9 ns (66.83%), attributed to excimer emission, which usually presents longer lifetimes compared to the monomeric species [34]. It agrees with the intensity inversion of the vibronic absorption bands and PL redshift in the spin-coated film compared to the solution. The intensity inversion suggests formation of H-aggregates, leading to excimer fluorescence [35]. Due to the similarity to the lifetime in solution, the 6.90 ns lifetime in the film is attributed to monomeric emission. The shortest lifetime of 0.90 ns can be related to the emission of aggregated species driven by π–π interactions [36], contributing to almost 20% of the total emission of **1**. The longer lifetime of **2** in film (3.84 ns) can be attributed to the monomeric emission as it is similar to the value observed in solution, while the shortest lifetime (1.58 ns) of aggregated π–π species dominates with 75.38% of the total emission.

**Table 2:** Excited state lifetimes and relative amplitudes of **1** and **2** in chloroform solutions and spin-coated films at room temperature.

Comp.	λ_max_ (nm)^a^	τ_1_ (ns)	A_1_ (%)	τ_2_ (ns)	A_2_ (%)	τ_3_ (ns)	A_3_ (%)	χ^2^

solution^b^								
**1**	487	6.54 ± 0.04	100	–	–	–	–	1.063
**2**	589	4.14 ± 0.03	100	–	–	–	–	1.008
film								
**1**	542	25.90 ± 0.2	66.83	6.90 ± 0.50	13.18	0.90 ± 0.20	19.99	0.997
**2**	633	3.84 ± 0.05	24.62	1.58 ± 0.03	75.38	–	–	0.946

^a^Fluorescence decay collected at maximum emission (λ_max_), excited at 401 nm. ^b^Concentration of 0.17 g L^−1^.

Hole-only (ITO/PEDOT: PSS/**1** or **2**/Au) and electron-only devices (Al/**1** or **2**/Al) were fabricated to evaluate the positive and negative charge carrier transport of **1** and **2**. The active layers of **1** or **2** were spin-coated from chloroform solutions (10 mg/mL). Scheme S1 in Supporting Information File 1 illustrates the device structure with the energy levels of the layers.

**Figure 5:**
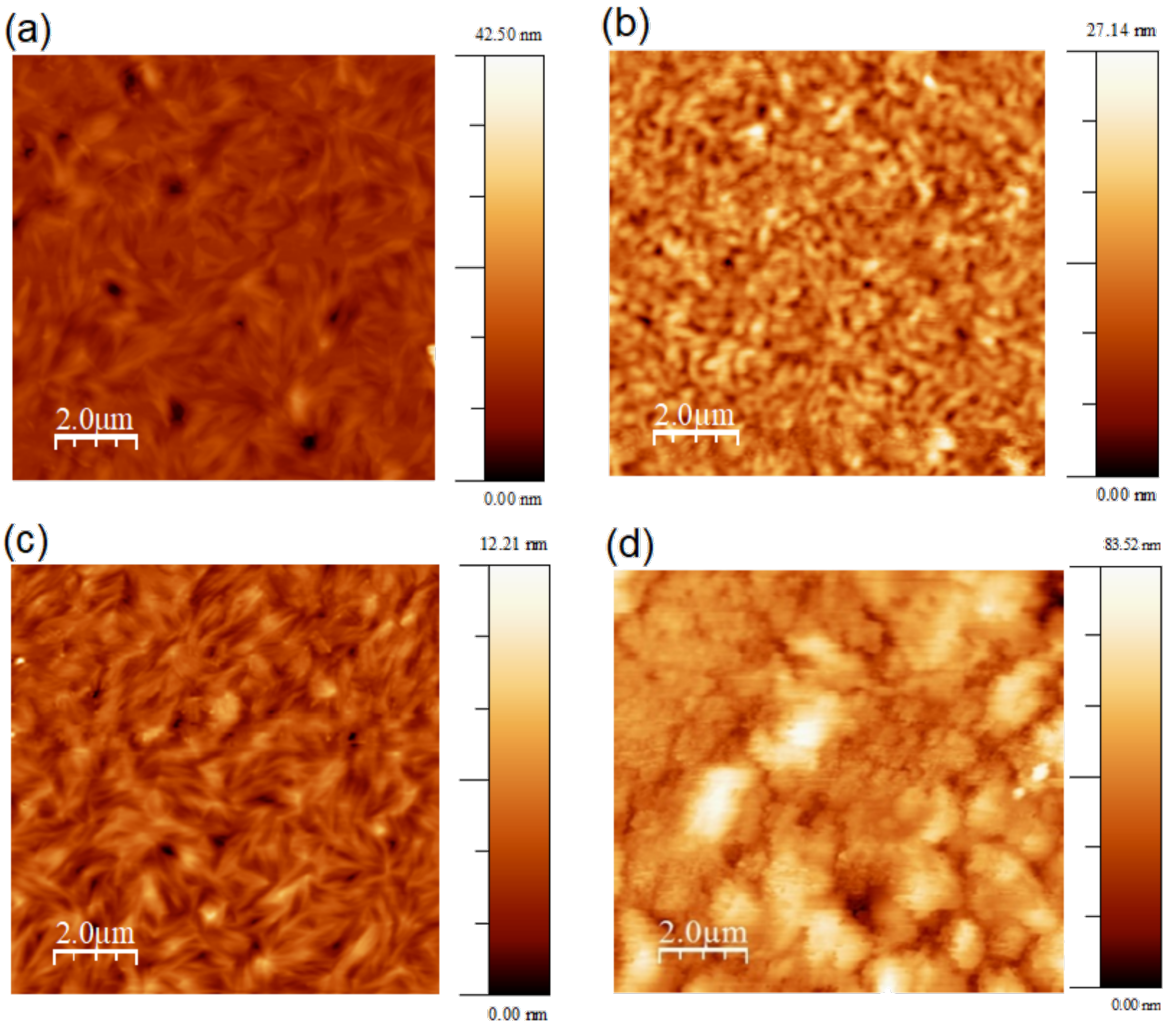
AFM images of spin-coated films of compound **1** (a, c) and compound **2** (b, d) on PEDOT:PSS (a, b) and Al (c, d).

Figure 5 shows atomic force microscopy (AFM) images to address the thickness and the morphology of the films of **1** and **2** deposited on the hole-only and electron-only device structures. The thickness of the films in these structures was 40 nm.

The films deposited on PEDOT:PSS for the hole-only devices indicate homogenous properties with low root mean square roughness (*R*_rms_) of 1.7 nm and 2.8 nm for **1** (Figure 5a) and **2** (Figure 5b), respectively. The *R*_rms_ of the films deposited on Al for the electron-only devices are 1.1 nm for **1** (Figure 5c) and 16.0 nm for **2** (Figure 5d). XRD measurements of as-casted spin-coated films of **1** and **2** confirm their columnar order and polarized optical microscopy show small birefringent domains, indicating locally instead of macroscopic molecular alignment of the films (see Figure S2 in Supporting Information File 1).

**Figure 6:**
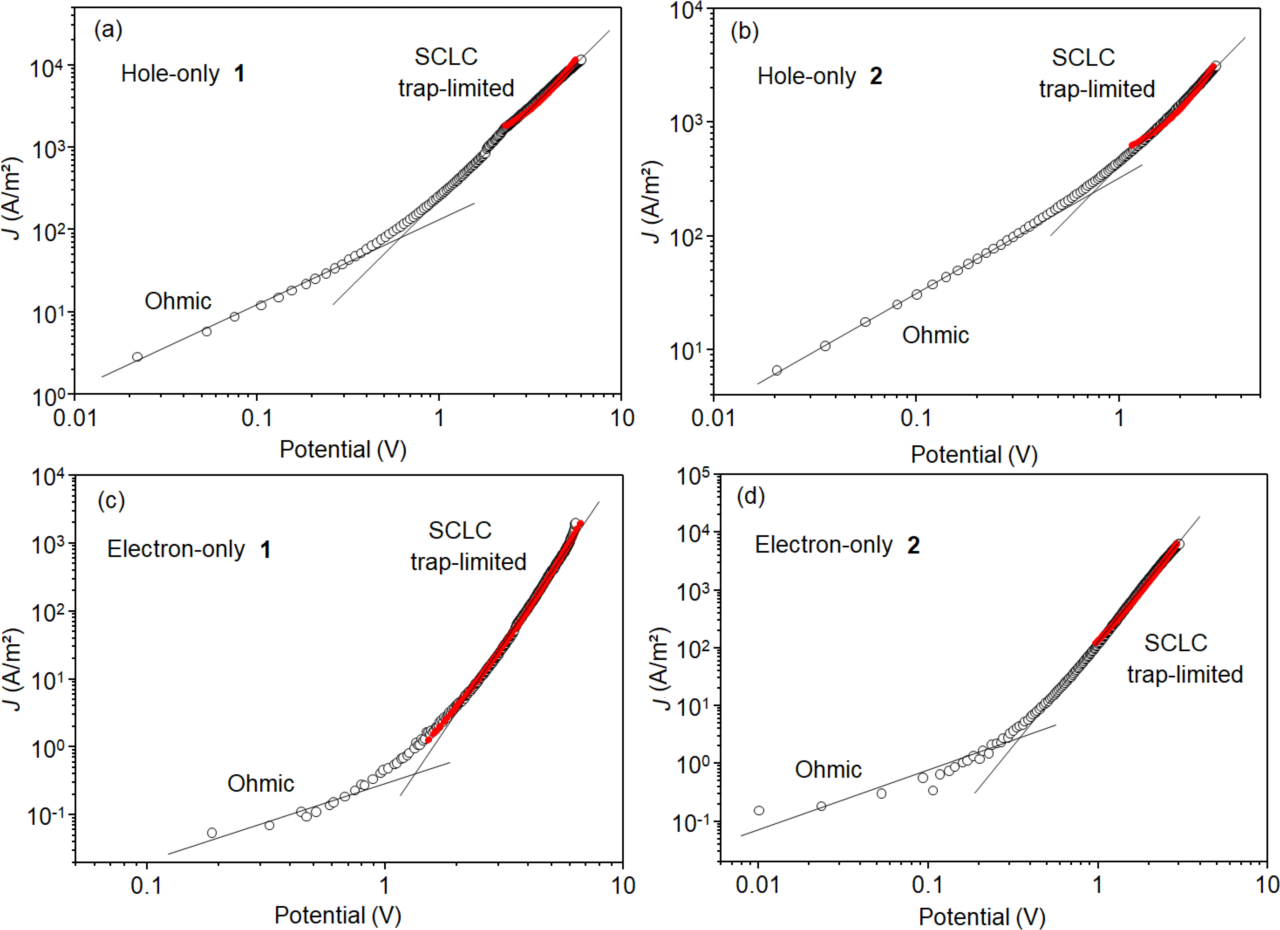
Log–log plot of the *J*–*V* curves of the hole-only (a,b) and electron-only (c,d) of **1** (a,c) and **2** (b,d). The open circles are the experimental data, and the red solid lines indicate the theoretical fitting. *V*_Ω_ is the threshold voltage between the ohmic and SCLC trap-limited regimes.

Figure 6 shows the log–log plots of the *J*–*V* experimental curves for **1** and **2** in both device structures. In all cases, an ohmic regime is observed at low voltages, 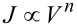 with *n* ≈ 1.0, followed by a space charge limited current (SCLC) trap-limited regime. To determine the charge carrier mobility, a previously published theoretical model was used [33], where an electric field dependent mobility of the form 
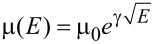
 was considered. The parameters µ_0_ and γ implicitly include a monoenergetic trap distribution and can be obtained from fitting the SCLC regimes of the experimental *J–V* curves. The fits for each device are shown as red solid lines in Figure 6, and the values of µ_0_ and γ displayed in Table 3 were used to calculate the mobility μ(*E*) at an electric field of 6.0 × 10^5^ V/cm.

**Table 3:** Fitting parameters and charge carrier mobility of hole-only and electron-only devices fabricated with **1** and **2**.

Comp.	γ (cm V^−1^)^1/2^	μ_0_ (cm^2^ V s^−1^)	μ (cm^2^ V s^−1^)^a^	Slope SCLC

hole-only				
**1**	2.49 × 10^−3^	5.82 × 10^−7^	1.2 × 10^−5^	2.1
**2**	3.18 × 10^−3^	6.62 × 10^−7^	2.7 × 10^−5^	1.8
electron-only				
**1**	7.18 × 10^−3^	2.54 × 10^−10^	6.4 × 10^−7^	5.2
**2**	5.74 × 10^−3^	2.24 × 10^−7^	1.3 × 10^−4^	3.6

^a^Mobility values for an electric field of 1.0 × 10^6^ V cm^−1^. Films of **1** and **2** were 40 nm thick.

**Figure 7:**
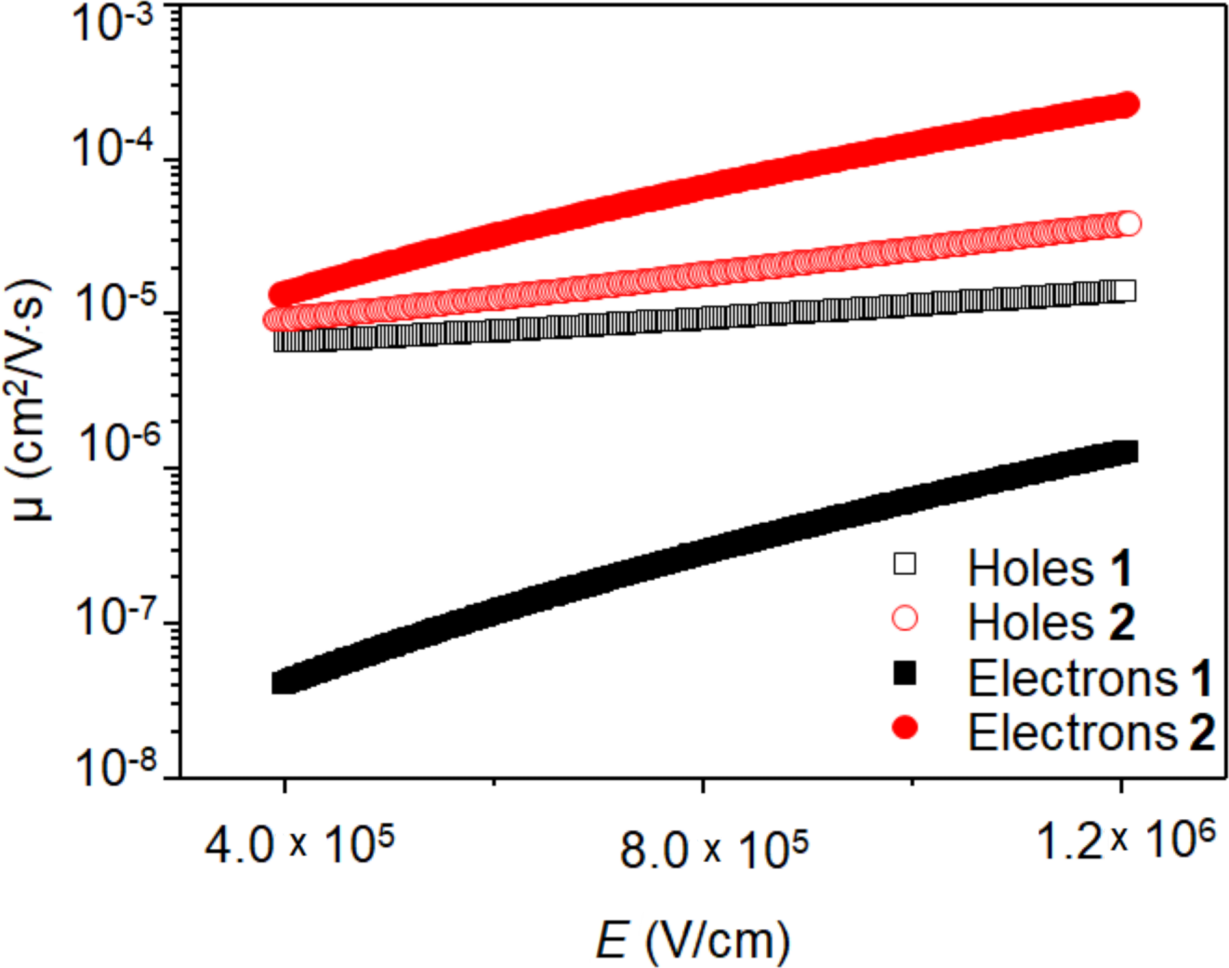
Charge carrier mobility as a function of the applied electric field obtained for the hole-only and electron-only devices of compounds **1** and **2**.

Figure 7 shows the charge carrier mobility as a function of the applied electric field, calculated from 
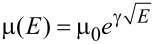
. The electron mobility of **1** is around one order of magnitude lower than that of holes, depending on the electric field. It suggests that this perylene-based material acts better as a hole-transporting material. The electron mobility of **2** is almost two orders of magnitude higher than **1**. It may be related to the closer molecular packing of the Col_rect_ phase preserved in the crystal at room temperature, which improves the π-stacking and corroborates with the dominant emission of aggregated π–π species in the film observed in the lifetime measurement. The hole mobility of **2** is similar to **1**, but its electron mobility is slightly higher, indicating that this pyrene-based molecule can act as an ambipolar transporting layer, in agreement with the literature [24]. It is important to emphasize that the charge carrier mobility of columnar liquid crystals can be improved by five orders of magnitude due to molecular alignment of the film in the device structure [33].

**Figure 8:**
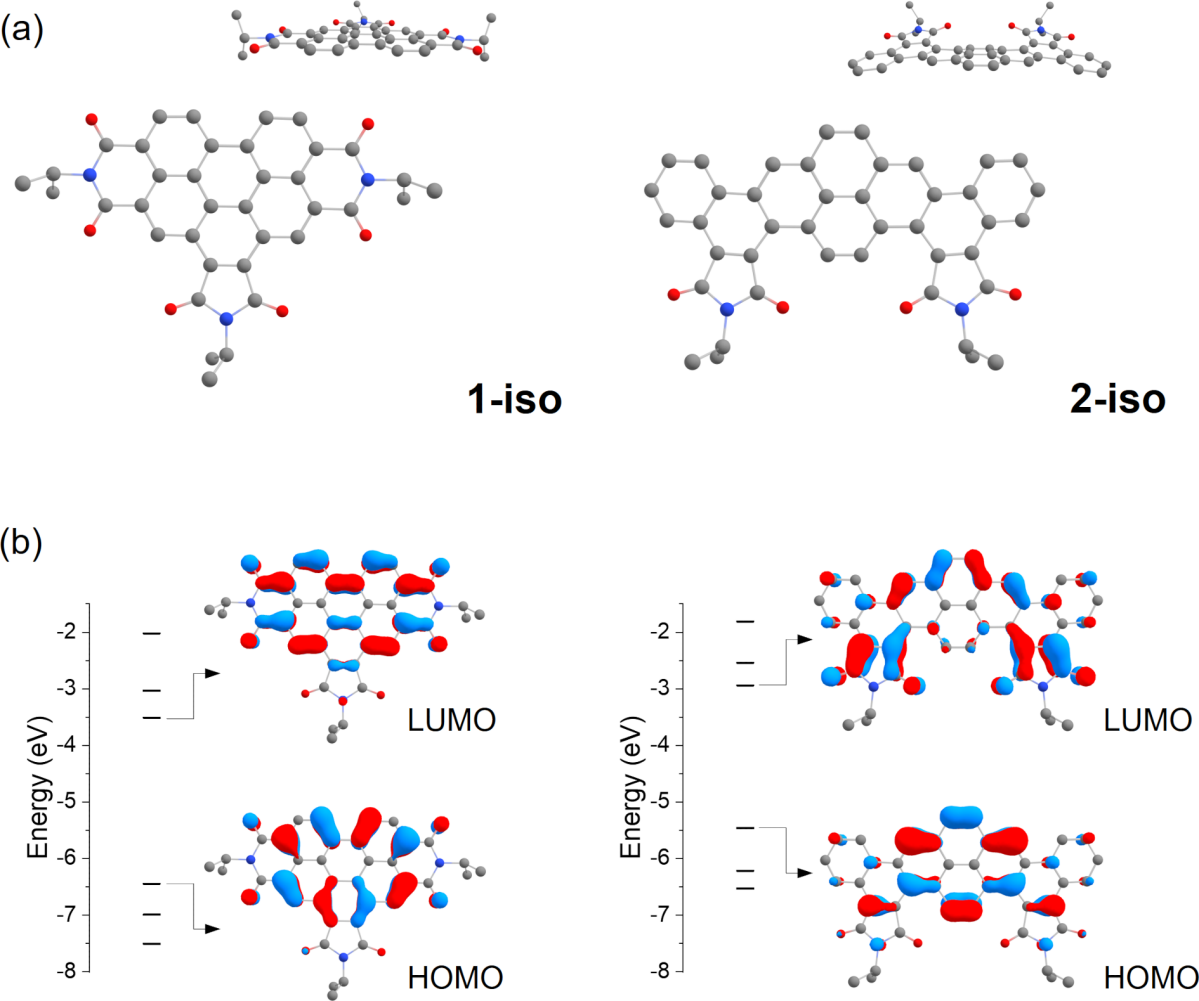
Ground state geometry (a) of compounds **1-iso** and **2-iso** obtained within B3LYP/def-TZVP(-f) level of theory and their frontier orbitals (b).

Usually, the π-system conjugation and the frontier orbitals energies dominates the charge transport [37–38]. Therefore, to better understand the charge mobility of compounds **1** and **2**, we obtained their ground state geometry using DFT within the B3LYP/def2-TZVP(-f) level of theory (Figure 8a). To reduce computational efforts, isopropyl moieties were used instead of the large aliphatic substituents of molecules **1** and **2**. The π electron systems of both molecules are predominantly planar, with **2-iso** showing a slight deviation from planarity due to the sterical proximity of the dicarboximide oxygens with the adjacent bay hydrogens. The frontier orbitals and their energies are shown in Figure 8b. In **1-iso**, only the two hexagonal imide groups affect the HOMO/LUMO localization, whereas the pentagonal central imide group is little affected. In **2-iso**, both pentagonal are relevant to the HOMO/LUMO localization, whereas the two terminal benzene rings are little affected. The cartesian coordinates of the optimized isopropyl derivatives geometry are presented in Supporting Information File 1.

## Conclusion

The electron and hole-transporting characteristics of two columnar liquid crystals were evaluated with electron-only and hole-only devices. Compound **1** is a perylene-based molecule presenting a columnar hexagonal mesophase stable at room temperature, and compound **2** is pyrene-based crystalline with columnar rectangular order preserved at room temperature. Photoluminescence lifetime measurements indicated that molecular aggregation and π-stacking interactions dominate the photophysics of films. While **1** presents majority hole transport, **2** is moderately ambipolar. The electron transport of **2** is around two orders of magnitude higher than **1**, which can be associated to the larger conjugation of **2**. This work highlights the potential of pyrene-based columnar liquid crystals as electron-transporting materials.

## Experimental

Raman spectra were obtained in an Anton Paar spectrometer, Cora 5001 model, under an excitation laser line at 785 nm and 450 mW power source. The exposure time was around 160 ms and 1 accumulation. The sample powder was placed in a glass slide under the objective lens and performed the autofocus.

XRD measurements were performed with an X’Pert PRO (PANalytical) diffractometer using Cu Kα radiation (λ = 1.5418 Å) equipped with an X’Celerator detector. A small amount of **1** or **2** was deposited onto a glass substrate and heated to the isotropic liquid phase. The diffracted radiation was collected in continuous mode from 2° to 30° (2θ angle) at specific temperatures during the cooling down to room temperature. The temperature was controlled with a TCU2000-temperature control unit (Anton Paar).

The absorbance spectra in solution and in spin-coated films were collected with an Ocean Optics USB4000 spectrophotometer. The PL spectra were collected with a Hitachi fluorescence spectrophotometer (Model F-7000) and the samples were excited at the wavelength of maximal absorption. For the PL as a function of the temperature, casting films were placed on a hot stage (Mettler Toledo FP-82) and excited with a UV lamp of 365 nm wavelength. The emission spectra were captured using an optical fiber placed close to the film to guide the light until the Ocean Optics USB4000 spectrophotometer.

Time-resolved fluorescence decay curves were recorded using the technique of time-correlated single photon counting [39] with a FluoTime 200 (PicoQuant). Excitation was provided using a 401 nm pulsed diode laser with repetition rates varying from 5.0 to 20 MHz. Fluorescence was collected perpendicular to excitation and passed through a polarizer set at the magic angle. The detection system consisted of a monochromator and a multichannel bases photomultiplier (Hamamatsu R3809U-50). Lifetimes were obtained by fitting the fluorescence decay curves with exponential functions using FluoFit^®^ software and the plots of weighted residuals and reduced chi-square were used to determine the best fits during the analysis procedure.

AFM measurements of the organic films were performed using a Nanosurf EasyScan2 apparatus in tapping mode with a scanning rate of 1.0 Hz covering 512 × 512 lines.

Hole-only devices were fabricated using indium tin oxide (ITO) coated glass plates (sheet resistance of about 15 Ω/sq) with a thin layer of PEDOT:PSS (purchased from Sigma-Aldrich) deposited by spin coating at 3000 rpm during 30 s, followed by annealing at 110 °C for 5 min as anodic electrode. For the electron-only devices, the anodic electrode consisted of a 100 nm thick Al layer vacuum deposited onto a glass substrate, 10^−7^ mbar at a rate of 2 Å/s. Compounds **1** and **2** were spin-coated from chloroform solutions (10 mg/mL) at 2000 rpm for 30 s, followed by annealing at 40 °C for 10 min. The top electrodes were vacuum deposited (10^−7^ mbar at a rate of 2 Å/s, 100 nm) onto the **1** and **2** layers, Au (for hole-only) and Al (for electron-only). The active area of the devices was 8 mm^2^. The *J–V* curves were measured at room temperature (25 °C) inside a glove box under nitrogen atmosphere using Keithley’s Series 2400 Source Measure Unit (SMU) instruments.

Ground state geometries of the molecules were optimized in a vacuum, using the Orca 5.0.3 [40] software package within B3LYP/Def2-TZVP(-f) level of theory [41–45]. Dispersion effects were included using Grimme’s D3 correction with Becke-Johnson (BJ) damping [45–46]. The evaluation of the four-center integrals was accelerated with the RIJCOSX algorithm [47–48]. RIJ requires the specification of an auxiliary basis set for the Coulomb part (Def2/J) and a numerical integration grid for the exchange part (DefGrid-2) [49]. Analytical harmonic vibrational frequency calculations were conducted to verify if the ground state is a minimum on the potential energy surface. Images of the complex geometries were obtained using the Chemcraft program (http://www.chemcraftprog.com).

## Supporting Information

File 1 Additional data and information.
