# Supplementary material for: Charge carrier transport in perylene-based and pyrene-based columnar liquid crystals
Source: Beilstein J Org Chem. 2023 Nov 16;19:1755–65. doi: 10.3762/bjoc.19.128 (PMC10667716; doi:10.3762/bjoc.19.128)
Supplement: File 1 — Additional data and information. [file Beilstein_J_Org_Chem-19-1755-s001.pdf]

BEILSTEIN JOURNAL OF ORGANIC CHEMISTRY

Supporting Information

for

Charge carrier transport in perylene-based and pyrene-based columnar liquid crystals

Alessandro L. Alves, Simone V. Bernardino, Carlos H. Stadtlober, Edivandro Girotto, Giliandro Farias, Rodney M. do Nascimento, Sergio F. Curcio, Thiago Cazati, Marta E. R. Dotto, Juliana Eccher, Leonardo N. Furini, Hugo Gallardo, Harald Bock and Ivan H. Bechtold

Beilstein J. Org. Chem. **2023,** *19,* 1755–1765. doi:10.3762/bjoc.19.128

Additional data and information

License and Terms: This is a supporting information file under the terms of the Creative Commons Attribution License (https://creativecommons.org/licenses/by/4.0). Please note that the reuse, redistribution and reproduction in particular requires that the author(s) and source are credited and that individual graphics may be subject to special legal provisions.
The license is subject to the *Beilstein Journal of Organic Chemistry* terms and conditions: (https://www.beilstein-journals.org/bjoc/terms)

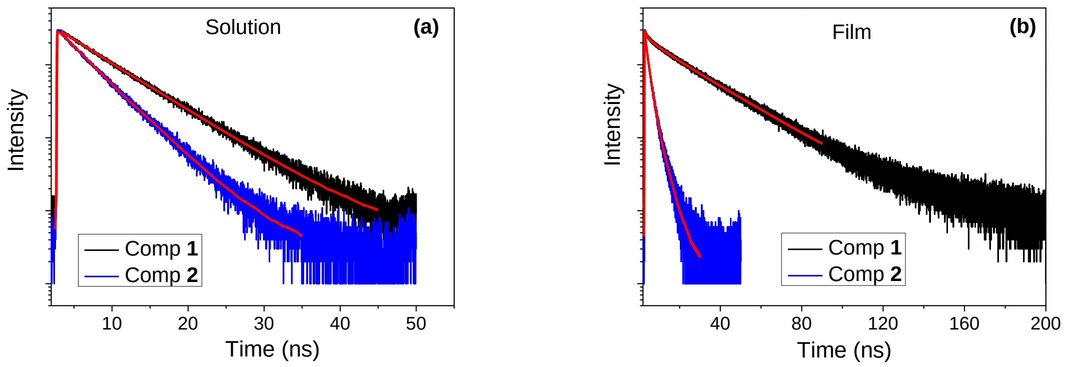

Figure S1: Fluorescence decay curves of compounds **1** and **2** in solution (a) and film (b). The red lines indicate the exponential fittings.

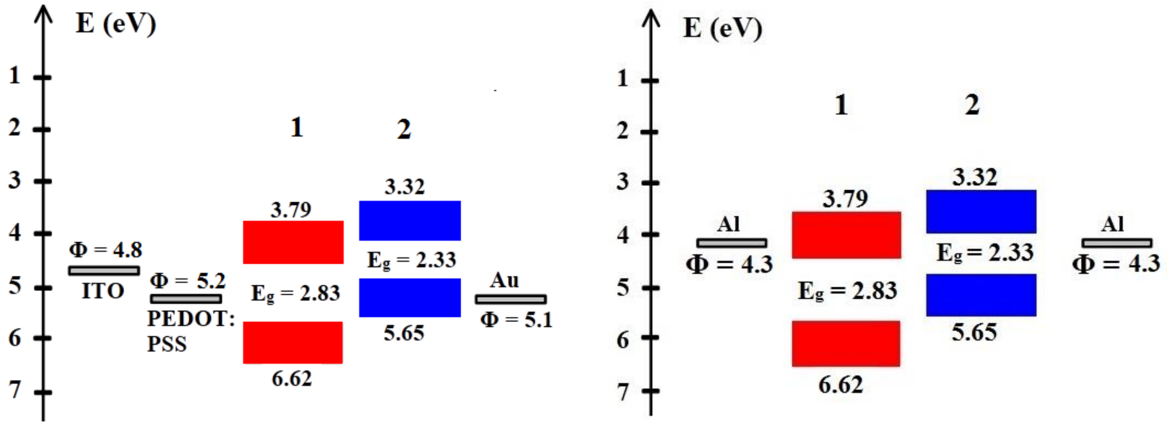

Scheme S1: Hole-only (left) and electron-only (right) device structures for compounds **1** and **2**.

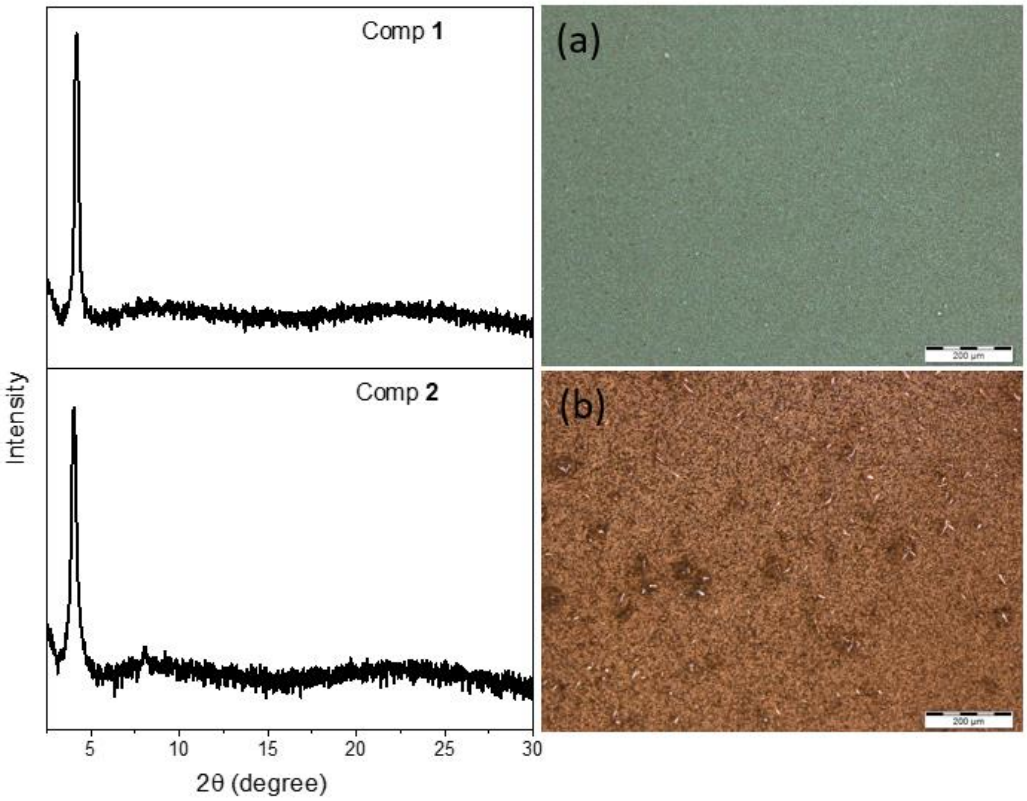

Figure S2: XRD and polarized optical microscopy of spin-coated films of **1** (a) and **2** (b).

Table S1. Calculated Cartesian coordinates for **1-iso**.

C	-3.58315748550623	0.96426507758844	-0.01127730929482
C	-4.60543668588654	-0.07658921177600	0.10459544787442
C	-4.19520735762568	-1.43090073279635	0.29648552614612
C	-2.20622002019265	0.59407939581935	0.06891553657631
C	-1.81154517881474	-0.76771798322179	0.26363446275810
C	-2.81105788254334	-1.78485469371254	0.37823174622915
C	-3.89851548441123	2.32172277510463	-0.20001822789941
C	-1.19792538007709	1.59549675205106	-0.04638374144486
C	-5.17830359135413	-2.45727024003410	0.40993481159026
C	-0.42183343985661	-1.09477437856802	0.34076289541672
C	-2.44404144030265	-3.15265308827426	0.57291441270607
C	0.56296799758017	-0.06778456843243	0.22063311672111

C	0.18488919038681	1.23800598750285	0.03158679647061
C	-1.55717584952266	2.94026590352043	-0.23782254310882
C	-0.09538818837993	-2.45720142839151	0.53594109644832
C	-1.06108553332103	-3.43901383909737	0.64661850015716
C	1.25247090112685	-3.10756495937041	0.65932592346298
C	-0.36999711844236	-4.75457723777505	0.84421314186689
C	-4.78064730553173	-3.81765711472178	0.60309979079945
C	-3.45321688754902	-4.15656105877964	0.68284725101825
C	-6.54437928511514	-2.13822026505233	0.33283918210160
C	-6.93582856122438	-0.81079079249351	0.14569708978196
C	-5.98271895920313	0.19889033220405	0.03414761062729
C	-7.58777492573148	-3.19160149178036	0.44742599950999
H	-8.00261579823737	-0.58888736953113	0.08976716029239
C	-2.90633461500727	3.29294745581715	-0.31162872292088
H	1.62107479815667	-0.32399371393412	0.27822051024047
C	1.24396243201482	2.28216444441826	-0.09407282352955
C	-0.52369616444634	4.00159948512787	-0.36616288848461
H	-3.15903939410906	4.34407218017607	-0.45894645000440
H	-4.94000261700419	2.63573019253765	-0.26196334767409
H	-3.16697766534542	-5.19820490257000	0.83039785918289
C	-5.80584734493778	-4.89573655020675	0.72182140342239
H	-6.32710991062499	1.22234037434599	-0.11106941834161
N	1.00347619883482	-4.47193750211162	0.84174152212309
C	2.03087304275917	-5.50957109517197	1.00974506114530
C	2.84524531481172	-5.26914498106491	2.28301551891551
H	3.42280136268393	-4.33623454460999	2.20901969439484
H	3.54748817166033	-6.10236548923569	2.44018606820259

H	2.18601850459641	-5.20732750768344	3.16273689386306
C	2.90043228727542	-5.62575112988325	-0.24429265773448
H	1.44817008820072	-6.43569049393860	1.12743223976622
H	3.47608627629919	-4.70318909499789	-0.40871727635085
H	2.28022161249437	-5.81858647147334	-1.13330032680889
H	3.60755725763769	-6.46213105507573	-0.13284169901740
N	-7.15012047146781	-4.51480573209163	0.63482755285958
C	-8.20448015779352	-5.56314017497984	0.74867303265785
H	-9.13425120951941	-4.99222251566792	0.64805014891430
C	-8.13475385329284	-6.56242389241093	-0.40760979218566
C	-8.19964807497523	-6.22100435248112	2.13006302290541
H	-8.25753200781521	-5.45954584276538	2.92369132896984
H	-7.29917039355295	-6.82943941639514	2.28652487157347
H	-9.08355221984742	-6.87149007888240	2.22340156667634
H	-7.23150432265796	-7.18418875679474	-0.35311875574253
H	-8.14809478117333	-6.03772616323928	-1.37598852085317
H	-9.01693482356829	-7.22080454771250	-0.37010359457899
N	0.82250948039662	3.60294602033841	-0.28995267132722
C	1.85015723043885	4.67397000229804	-0.43363030624315
H	1.24793348149778	5.57848680988858	-0.57381630158003
C	2.70209160105264	4.47297506295904	-1.68836208017801
C	2.67116836560899	4.84592084811549	0.84555998571136
H	2.01092715166563	4.99920447501612	1.71368859390041
H	3.30963227133224	5.73803253469100	0.74735327523244
H	3.31278287027587	3.97553206271376	1.03576865087696
H	2.06287890286710	4.36023471666210	-2.57824495680195
H	3.34994463357959	3.59073994612076	-1.60236961718896

H	3.33597880777662	5.36072320103725	-1.84052208029770
O	-0.83504073028541	5.16716347930077	-0.53232738800337
O	2.42486676439837	1.99532207729998	-0.03025808133656
O	-8.77186479271315	-2.91508387170165	0.38108925616614
O	-5.48453698802141	-6.05785032900515	0.88657473467176
O	-0.85649464911119	-5.85382630489053	0.98123685720176
O	2.34592254868998	-2.58798462787097	0.61498443080284

Table S2. Calculated Cartesian coordinates for **2-iso**.

C	-3.53755610986468	0.78161710826123	-0.22987129849116
C	-4.43610659850284	-0.21597944474441	-0.02579730512958
C	-4.00717818139385	-1.52721581129557	0.39114307623448
C	-2.12643464633481	0.55973386883416	-0.03804945671951
C	-1.65975256157212	-0.74485913729950	0.33593015529642
C	-2.60701663820962	-1.79566715528641	0.55444213536261
H	-3.86827370400224	1.77911577520191	-0.53035102846718
C	-1.21538937572966	1.59843431131864	-0.16217040936663
H	-5.50672661674333	-0.03975717379631	-0.15760760313907
C	-4.92871802258459	-2.52128143719210	0.68470074920324
C	-0.26492122755257	-0.96762027923621	0.51640231327260
C	-2.18153485486597	-3.09224194860562	0.96262797947675
C	0.67813618041310	0.09141344516198	0.22971246019800
C	0.17541474747182	1.41741792103197	-0.00991234958593
H	-1.60895877835472	2.58522838292626	-0.40071921898498
C	1.07426587322810	2.56637468657288	-0.09393472010031
C	0.10854255263643	-2.22752079156888	1.06747521144613

C	2.10686945886084	-0.06421198120367	0.15792482609846
C	-0.79925372957413	-3.23300905113284	1.28015960257450
H	1.14063075854388	-2.40957212836650	1.34093436288960
H	-0.43841559470119	-4.15890386929238	1.71037103572853
C	-4.55078285031676	-3.82251567013223	1.07699201261581
C	-3.15186582214567	-4.15468783078676	1.12002852089823
C	-2.84039672935189	-5.54619443251427	1.31859086847151
C	2.48744201355404	2.37177559257334	-0.02737400169852
C	2.94705804636865	1.02595241429458	0.04810476676544
C	2.98099653064244	-1.29279816020592	-0.00965723581952
C	4.36452918647845	0.55507119436425	-0.05472482259610
H	-5.98413538415273	-2.27063202606914	0.59143544493290
C	-5.57573711971298	-4.80044190521135	1.43479884316460
C	0.60298387465839	3.89717015461274	-0.21613585238315
C	3.37079672603488	3.48085229317849	-0.08247130917390
C	-3.82138970039169	-6.46931563798155	1.62325227679480
C	-1.56858280809864	-6.34094622483605	1.08729627716735
C	-5.20208959354512	-6.14275511524870	1.74636869670851
C	-6.18513018288960	-7.09606703197519	2.11835156978682
C	1.47711835582235	4.96931785138501	-0.26549527762034
C	2.87015462275428	4.76310619182026	-0.19910952817513
H	4.44241360695974	3.29101267762547	-0.03523030719223
H	3.55170159678591	5.61613946284626	-0.23848365516226
H	-0.46645104542204	4.09934409829965	-0.25623953143431
H	1.08011125797717	5.98380229095537	-0.35175794301875
C	-3.21038125944811	-7.83240573679842	1.72663245319517
C	-7.51591688480428	-6.73198564608054	2.18666811012811

C	-7.89811225032999	-5.40926914965377	1.88403547505929
C	-6.95122647759497	-4.46935947088451	1.51551253638222
H	-8.94982279234658	-5.11865886187452	1.94492455748925
H	-7.28491746396164	-3.45428338387250	1.30479597363972
H	-5.86186865352098	-8.11197509095571	2.34191740242758
H	-8.27077971842970	-7.46668032066388	2.47663381487338
N	-1.86856380924950	-7.67418326326037	1.39783219457427
N	4.30339811330178	-0.83401056555574	-0.08211889619849
O	-3.74157971964062	-8.88419996151351	2.02088597525039
O	-0.48689949643230	-5.98934391751008	0.67626882291206
O	2.68179607680599	-2.45972777697087	-0.11619391544979
O	5.38268676740150	1.21424883738726	-0.11616303844457
C	-0.88088897652539	-8.75538770291259	1.29133852926866
C	5.44970448977836	-1.73566466528578	-0.25086462300420
C	-0.66895250590982	-9.43641858555401	2.64525967553905
H	0.04425471860745	-8.23542991212862	1.00186422770910
C	-1.26035921052692	-9.73829848359946	0.18119804595801
H	-2.20193131924571	-10.25494808149109	0.41869505678333
H	-0.46863983240914	-10.49442159861121	0.06253831118207
H	-1.37816634662153	-9.21413234553405	-0.77991552755603
H	-0.37886031020084	-8.70036984671532	3.41113621422671
H	0.13580725898359	-10.18384843119411	2.56657598312143
H	-1.58648587699629	-9.94410773215650	2.97616583235659
C	6.41627560000987	-1.61573950198645	0.92973174060319
C	6.12912858162264	-1.51216657376008	-1.60427166406304
H	4.99648742637121	-2.73801101169046	-0.24378642503362
H	6.57029035706242	-0.50623509696933	-1.66079051105699

H	5.40595057476197	-1.62724788120074	-2.42654163968185
H	6.93111702638051	-2.25286089675867	-1.74831818950576
H	6.86436195046135	-0.61235472109584	0.96888777966497
H	7.22479648152640	-2.35629542393104	0.82798204147882
H	5.89585996794228	-1.80576267650014	1.88137334534221
